# Glycines from the APP GXXXG/GXXXA Transmembrane Motifs Promote Formation of Pathogenic Aβ Oligomers in Cells

**DOI:** 10.3389/fnagi.2016.00107

**Published:** 2016-05-10

**Authors:** Marie Decock, Serena Stanga, Jean-Noël Octave, Ilse Dewachter, Steven O. Smith, Stefan N. Constantinescu, Pascal Kienlen-Campard

**Affiliations:** ^1^CEMO-Alzheimer Dementia, Institute of Neuroscience, Université Catholique de LouvainBrussels, Belgium; ^2^Department of Biochemistry and Cell Biology, Stony Brook University, Stony BrookNY, USA; ^3^Ludwig Institute for Cancer Research – de Duve Institute, Université Catholique de LouvainBrussels, Belgium

**Keywords:** Alzheimer’s disease, amyloid precursor protein, beta-amyloid peptide, oligomers, GXXXG motifs, neuronal differentiation

## Abstract

Alzheimer’s disease (AD) is the most common neurodegenerative disorder characterized by progressive cognitive decline leading to dementia. The amyloid precursor protein (APP) is a ubiquitous type I transmembrane (TM) protein sequentially processed to generate the β-amyloid peptide (Aβ), the major constituent of senile plaques that are typical AD lesions. There is a growing body of evidence that soluble Aβ oligomers correlate with clinical symptoms associated with the disease. The Aβ sequence begins in the extracellular juxtamembrane region of APP and includes roughly half of the TM domain. This region contains GXXXG and GXXXA motifs, which are critical for both TM protein interactions and fibrillogenic properties of peptides derived from TM α-helices. Glycine-to-leucine mutations of these motifs were previously shown to affect APP processing and Aβ production in cells. However, the detailed contribution of these motifs to APP dimerization, their relation to processing, and the conformational changes they can induce within Aβ species remains undefined. Here, we describe highly resistant Aβ42 oligomers that are produced in cellular membrane compartments. They are formed in cells by processing of the APP amyloidogenic C-terminal fragment (C99), or by direct expression of a peptide corresponding to Aβ42, but not to Aβ40. By a point-mutation approach, we demonstrate that glycine-to-leucine mutations in the G^29^XXXG^33^ and G^38^XXXA^42^ motifs dramatically affect the Aβ oligomerization process. G33 and G38 in these motifs are specifically involved in Aβ oligomerization; the G33L mutation strongly promotes oligomerization, while G38L blocks it with a dominant effect on G33 residue modification. Finally, we report that the secreted Aβ42 oligomers display pathological properties consistent with their suggested role in AD, but do not induce toxicity in survival assays with neuronal cells. Exposure of neurons to these Aβ42 oligomers dramatically affects neuronal differentiation and, consequently, neuronal network maturation.

## Introduction

The amyloid precursor protein (APP) is a ubiquitously expressed type 1 transmembrane protein (Kang et al., 1987; Selkoe, 2004) whose processing in the amyloidogenic pathway leads to the production of the β-amyloid peptides (Aβ). Aβ peptides are the major constituent of the senile plaques, a hallmark of AD (Glenner and Wong, 1984). Mutations responsible for inherited AD cases (early onset or familial AD) are located in the genes coding for APP or the presenilins (Kandimalla et al., 2012). The presenilins (PS1 and PS2) are the catalytic subunits of γ-secretase, a multiprotein complex that cleaves the APP β-C-terminal fragment (βCTF or C99) to generate Aβ in the last step of the amyloidogenic pathway. APP or PS mutations typically result in imbalanced Aβ production and an increased Aβ42/Aβ40 ratio (Selkoe, 2004). These observations led to the amyloid cascade hypothesis, predicting that the initial steps of AD, which trigger a series of pathogenic events, are related to Aβ production and clearance (Hardy and Allsop, 1991). Although this hypothesis remains a matter of debate (Herrup, 2015), experimental lines of evidence from cellular models, transgenic animals and patient brain samples has been overwhelming (Hardy, 2009). Less is known about the cellular mechanisms that control the formation of pathological Aβ oligomers. Both cellular trafficking, particularly endocytosis, and posttranslational modification like phosphorylation have been involved in Aβ production and accumulation (Feyt et al., 2005, 2007).

Alzheimer’s disease onset and progression appears to be directly linked to the accumulation of abnormally folded Aβ assemblies. Although fibrillogenic species were first suggested to be responsible for AD-induced neurotoxic events, growing evidence shows that soluble Aβ oligomers are more strongly correlated with clinical symptoms associated with the disease (Lesne et al., 2006; McDonald et al., 2010; Kandimalla et al., 2011, 2014). Different Aβ oligomers have been described, ranging from dimers to dodecamers and high molecular weight assemblies. These assemblies can be classified between an “off-pathway” or an “on-pathway” with respect to fibrillization. Soluble oligomers have been characterized in transgenic mouse brains (Kawarabayashi et al., 2001; Lesne et al., 2006; Shankar et al., 2009; Pham et al., 2010), and to some extent in brains of AD patients (Hayden and Teplow, 2013). Still, the key toxic Aβ species remain poorly defined in terms of both molecular structure and relevance to the mechanisms underlying long term potentiation defects and neuronal cell death.

Structural studies on Aβ assemblies have largely come from *in vitro* measurements of synthetic peptides corresponding to Aβ40 and Aβ42. Aβ40 is the predominant isoform (∼90%) generated by γ-secretase cleavage, while Aβ42 (10%) is the major component of amyloid plaques. Monomeric Aβ adopts predominantly a random coil structure. Monomers associate into small MW oligomers (dimers – hexamers) that are able to combine into larger MW oligomers, which in turn laterally associate into protofibrils (Fu et al., 2015). The conversion of protofibrils to fibrils involves a transition to cross-β-structure. The conversion implies association of the short hydrophobic LVFF sequence with the hydrophobic C-terminus of Aβ (Fu et al., 2015).

Glycines appear to be important in both the turn region between β-strands and in the C-terminal β-sheet. Glycines have a critical impact on peptide aggregation, facilitating the association of β-sheets during fibril formation *in vitro* (Liu et al., 2005; Sato et al., 2006). Fibrillization of synthetic Aβ peptides containing glycine-to-leucine (G to L) mutations has been monitored *in vitro* (Kim and Hecht, 2006; Hung et al., 2008). Treatment of neuronal cells showed a reduction of toxicity for mutated peptides when compared to non-mutated Aβ. Reduced toxicity correlated with a reduction of small oligomeric species in solution and increased rates of fibril formation (Hung et al., 2008). Using synthetic peptides, Harmeier et al. (2009) highlighted G33 as critical for the generation of Aβ42 assemblies. Mutation of G33 promoted rapid Aβ oligomerization by conformational changes that favored the formation of high molecular weight oligomers, which were less pathogenic than Aβ42. In contrast, a G37L substitution dramatically reduced Aβ toxicity as measured by cell dysfunction, cell death, synaptic alteration in primary neurons and transgenic *Caenorhabditis elegans* models (Fonte et al., 2011). Important limitations in studies using synthetic Aβ peptides to generate oligomers *in vitro* are their exact relevance to AD pathology. The soluble oligomers are formed *in vitro* at relatively high concentrations. At lower concentrations, which may be more representative of physiological conditions, the monomer – oligomer equilibrium shifts toward the monomeric state, which is non-toxic and presumably is more easily cleared from the brain. The structure and stability of soluble oligomers that are produced physiologically have consequently been of considerable interest.

G33 and G37 lie within the hydrophobic C-terminus of the Aβ peptide and represent the third APP TM GXXXG motif. GXXXG motifs occur abundantly in the TM helices of membrane proteins where they facilitate TM helix dimerization and close apposition. Strikingly, C99 has three consecutive GXXXG motifs, followed by a GXXXA motif, all of which have been implicated in dimerization and regulation of C99 processing by the γ-secretase complex (Munter et al., 2007; Kienlen-Campard et al., 2008). The structural role that the GXXXG motif plays critically depends on the structural element in which it is located and the exposure to water. In aqueous environments, glycine is able to adopt multiple conformations due to the lack of a side chain and the ability of the backbone NH to hydrogen bond to water (glycine is known as a helix breaker in soluble proteins and often occurs in turn sequences). In hydrophobic TM helices, glycines in a GXXXG motif fall on one face of a TM α-helix and facilitate helix-helix dimerization. The TM helices are energetically favored due to intrahelical backbone hydrogen bonding in the absence of water and helix association is favored due to interhelical van der Waals and hydrogen bonding interactions. Interestingly, in β-sheets the glycines in a GXXXG motif lie on one face of the β-sheet and when the β-strands are parallel and in-register, they form long surface grooves that facilitate sheet-to-sheet packing that excludes water (Sato et al., 2006). This multifaceted nature of glycine is highlighted in its role at each stage of the process from TM helix to soluble oligomer to fibril. The same multifaceted nature of glycine may underlie the aggregation of the human prion protein, which also contains three consecutive GXXXG motifs.

We have further investigated the role of the GXXXG/GXXXA motifs in dimerization of C99 and oligomerization of Aβ produced by living cells. We found that mutation of the critical G33 and G38 residues, respectively, in the G^29^XXXG^33^ and G^38^XXXA^42^ motifs did not affect dimerization of C99. In contrast, mutations in these motifs triggered the assembly of Aβ42, in living cells, into ∼28 kDa oligomers corresponding to the expected molecular weight of Aβ hexamers. Similar oligomers are not detected with constructs producing only Aβ40. The Aβ42 oligomers generated by living cells are resistant to temperature and denaturing conditions. They are enriched in membrane-bounded compartments, and released in the extracellular medium. Finally, we showed that Aβ42 oligomers generated by living cells did not display neurotoxic effects, but greatly affected neuronal differentiation and the formation of neuronal networks.

## Materials and Methods

### Chemicals and Reagents

Restriction enzymes, Taq DNA polymerase, all culture media, penicillin-streptomycin solution, HAT and Lipofectamine^®^ transfection reagents, Nu-Page^®^ Novex^®^ 4–12% Bis-Tris gels and buffers were from Life Technology Corporation (Carlsbad, CA, USA). Fetal bovine serum (FBS) for culture media was purchased from Thermo Scientific (Rockford, IL, USA). Analytical grade solvents, salts and poly-L-lysine were from Sigma–Aldrich (St Louis, MO, USA). Protease inhibitor cocktail was purchased from Roche (Basel, Switzerland). BCA protein assay kit was from Pierce (Rockford, IL, USA). Nitrocellulose membranes were obtained from GE Healthcare (Fairfield, CT, USA). ECL reagents were obtained from Perkin Elmer Inc. (Waltham, MA, USA). The following primary antibodies were used: anti-Amyloid β Antibody, clone W0-2 (EMD Millipore, Billerica, MA, USA), Anti-Amyloid Precursor Protein C-terminal, anti-MAP2 and anti-actin antibody (Sigma–Aldrich, St Louis, MO, USA), and anti-GLuc antibody (New England Biolabs, Ipswich, MA, USA). Fluorescent nucleic acid stain DAPI was obtained from Sigma–Aldrich (St Louis, MO, USA). Secondary antibodies coupled to HRP were obtained from Amersham Bioscience (Uppsala, Sweden) and fluorescent secondary antibody coupled to Alexa fluorochromes from Life Technology Corporation (Carlsbad, CA, USA).

### Cells Lines and Cell Culture

Chinese hamster ovary (CHO) cell lines were grown in Ham’s F-12 medium. The media was supplemented with 10% of FBS and penicillin-streptomycin solution (10 units–10 μg). All cell cultures were maintained at 37°C in a humidified atmosphere (5% CO_2_). Mouse neuroblastoma × Rat glioma hybrid cell lines (NG108-15) were grown in DMEM supplemented with 10% FBS, 2% HAT, a mixture of hypoxanthine, aminopterin and thymidine, and antibiotics. Differentiation of NG108-15 cells was induced by switching from regular medium to 1% FBS medium (Stanga et al., 2015).

### Plasmids, Cloning, and Site-Directed Mutagenesis

C99, C42 mutants and various C-terminally truncated constructs of C99 were obtained by Quick-change site-specific mutagenesis (Stratagene, La Jolla, CA, USA) as previously described (Ben et al., 2012b). The plasmids expressing APP fragments fused to humanized *Gaussia* luciferase (hGluc) halves were obtained by PCR amplification of APP sequences encoded by expression vectors previously described (Decock et al., 2015). All constructs were verified by full sequencing (Macrogen Europe, Amsterdam, The Netherlands).

### Cell Transfection and Conditioning

Chinese hamster ovary cells were transfected with Lipofectamin reagent 24 h after seeding following manufacturer’s instructions. Plasmids expressing the split-luciferase proteins were cotransfected in a 1:1 ratio. The control plasmid (mock) used was the corresponding empty vector. 48 h after transfection, media were collected, treated with protease inhibitors cocktail (Roche) and stored at -20°C for ECLIA assay. Cells were harvested and lysed in sample buffer (125 mM TrisHCl pH 6.8, glycerol 20% and SDS 4%) supplemented with protease inhibitor cocktail. Protein concentrations were measured by the BCA protein assay kit from Pierce (Rockford, IL, USA) prior to Western blotting.

Transfected CHO cell culture media have been used as source of oligomers to treat NG108-15 cells at days 1 and 3 of differentiation. One day after transfection, CHO cells were maintained in medium without FBS. The CHO conditioned media enriched in oligomers were mixed (1:1 ratio) to NG108-15 cells differentiation medium (1% FBS). Treated NG108-15 cells were fixed and processed for immunocytochemistry (day 5).

### Western Blotting

Proteins (10 μg) from cell lysates or culture media (20 μl) were heated for 10 min at 70°C in loading buffer (lysis buffer supplemented with 0.5 M DTT and staining Nupage blue^TM^), separated in 4–12% Nupage^TM^ bis-Tris gel and transferred for 2 h at 30 V onto nitrocellulose membranes. Ponceau Red staining was used to check gel loading and transfer accuracy. After blocking (5% non-fat milk in PBS), membranes were incubated overnight at 4°C with the primary antibodies: anti-Amyloid β Antibody, clone W0-2 (1/2,500), Anti-Amyloid Precursor Protein, C-terminal antibody (1/2,000), anti-GLuc antibody (1/2,000). Membranes were washed with PBS-Tween (0.005%) and incubated with the secondary antibodies anti-mouse (1:10,000) or anti-rabbit (1:10,000) coupled to peroxidase prior to ECL detection from GE Healthcare (Little Chalfont, UK). Signals were quantified with a Gel Doc 2000 imaging system coupled to Quantity one^TM^ software from Bio-Rad (Hercules, CA, USA).

### *Gaussia* Luciferase Assay

Samples were aliquoted in 5 ml polystyrene round-bottom tubes at a final concentration of 10 μg of protein in 20 μl in Luciferase Cell Lysis Buffer (Promega, Madison, WI, USA). Native coelenterazine was reconstituted as a stock solution of 1 mg/ml in methanol (stored frozen), diluted 30 min prior reading in DMEM without phenol red and used at a final concentration of 20 μM. 50 μl of coelenterazine was added to tubes and luminescence directly measured on a Sirius Luminometer (Berthold, Pforzheim, Germany).

### Aβ Quantification

Aβ38, Aβ40, and Aβ42 peptides were quantified in the cell medium as previously described (Hage et al., 2015) using the Aβ multiplex electro-chemiluminescence immunoassay (ECLIA; Meso Scale Discovery, Gaithersburg, MD, USA). Cells were conditioned in serum-free medium for 16 h; cell medium was collected and Aβ were quantified according to the manufacturer’s instructions with the human Aβ specific 6E10 multiplex assay.

### Cell Survival Assay

Cell viability (NG108-15 cells) was assessed with a MTS assay after 24 h of treatment, according to manufacturer’s instruction (Promega, Madison, WI, USA). Plates were measured at 490 nm using a microplate spectrophotometer Victor X3 Multilabel Plate Reader (PerkinElmer, Waltham, MA, USA).

### Immunocytochemistry

Cells were seeded on 12-well plates previously incubated with poly-L-lysine (10 mg/ml). Prior to staining, cells were rinsed twice with Opti-MEM^®^ (Life Technology Corporation) and fixed with 4% paraformaldehyde (PFA) for 15 min. After three washes in PBS, cells were permeabilized with PBS1X/0.3% Triton100X for 30 min and blocked in PBS1X/fetal bovine serum 5%/0.1% Triton100X for 30 min. Primary antibody MAP2 (1:500) was prepared in the blocking solution and incubated O/N at 4°C. After three washes in PBS, cells were incubated with secondary antibody (Goat anti-mouse Alexa 488, 1:500 in blocking solution) and DAPI (1:2000) for 1 h at 4°C. After three washes in PBS, cells were stored in PBS-azide 0.1% at 4°C. Pictures were acquired with an Evos fluorescence microscope (Advanced Microscopy Group, Mill Creek, WA, USA).

### Statistical Analysis

The number of samples (n) in each experimental condition is indicated in figure legends. The data were analyzed using GraphPad Prism software by analysis of variance (ANOVA) followed by unpaired *t*-test (two experimental conditions) or by Bonferroni’s Multiple Comparison tests (more than two experimental conditions).

## Results

### Expression of APP C-Terminal Fragments Leads to the Accumulation of Oligomeric Aβ Peptides

In our previous work we reported that expression of C99 (corresponding to the β-CTF of APP) leads to the formation of an oligomer, which is detected in denaturating gels as a higher molecular weight band (around 25 kDa). This band was thought to contain dimers of C99 (Kienlen-Campard et al., 2008), but also -possibly- oligomers of Aβ or other peptides, as well as a mix of C99 and truncated C99 peptides.

**FIGURE 1:**
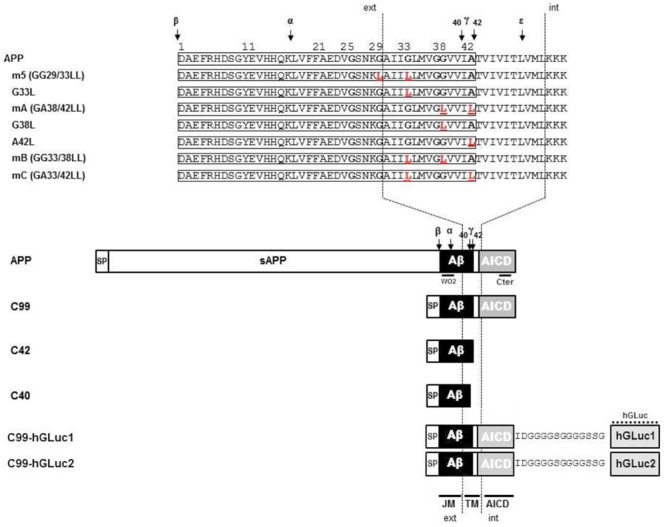
**Schematic representation of the different amyloid precursor protein (APP) mutants and APP constructs used for the study.** Schematic representation of human APP and APP C-terminal fragments including the different mutants generated. Numbering corresponds to the amino acid position in the Aβ sequence. C99 corresponds to the APP β C-terminal fragment, C42 to the Aβ42 peptide, and C40 to the Aβ40 peptide. All C-terminal constructs are fused to the human APP signal peptide (SP). TM, Transmembrane region; JM, Juxtamembrane region; AICD, APP IntraCellular Domain; ext, extracellular; int, intracellular. The amino acid substitutions generated by site-directed mutagenesis are in red. The cleavage sites of α (α)-, β (β)-, and γ (γ and ε)-secretases are indicated by arrows. C99-hGLuc1 and C99-hGLuc2 correspond to C99 constructs fused to hGLuc moieties used for the analysis of C99 dimerization in split luciferase assays. The epitopes recognized by the human-specific W0-2 antibody, the APP C-terminal and hGLuc antibodies are indicated.

In order to characterize the nature of this higher molecular weight band, we expressed in CHO cells different APP constructs: human APP695 (neuronal isoform of APP), C99 (the amyloidogenic β C-terminal stub) and C42, which corresponds to the sequence of human Aβ42, that we engineered by adding a stop codon after residue 42 of Aβ. Both C99 and C42 are fused to the APP signal peptide to ensure proper targeting to the secretory pathway. Similar constructs were generated by introducing a stop codon after residue 40 of Aβ to generate C40, or by fusing *Gaussia* luciferase moieties to the C-terminus of C99 to measure its dimerization by a split protein assay (Decock et al., 2015). All these constructs are depicted in **Figure 1**.

**FIGURE 2:**
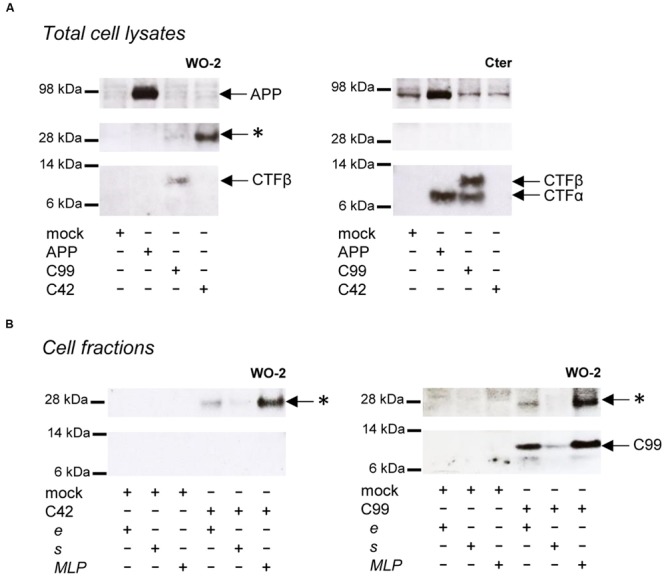
**Detection of oligomeric bands in cells expressing C99 and C42. (A)** Expression of APP, C99 and C42 in CHO cells analyzed in total cell lysates by Western blotting with the W0-2 antibody (left panel) and the APP C-terminal antibody (right panel). The presence of APP, βCTF (C99) and αCTF are indicated by arrows. The star (^∗^) indicates the presence of an unexpected band at ∼30 kDa. **(B)** Cell fractions from C42- (left panel) and C99- (right panel) transfected cells were analyzed by Western blotting with the W0-2 antibody. Total cell lysates (e) were fractionated into soluble (s) and membrane-enriched vesicular fractions (MLP). The presence of the higher molecular weight band (^∗^) is indicated by arrows.

**FIGURE 3:**
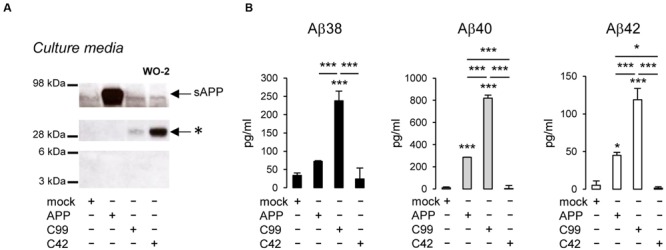
**Detection of oligomeric bands and measurement of Aβ in the extracellular medium of cells expressing C99 and C42. (A)** Culture media of CHO cells expressing APP, C99, and C42 were analyzed by Western blotting using the W0-2 antibody. The ∼30 kDa oligomer band (^∗^) detected in culture media -similar to the one observed in cell lysates- is indicated by an arrow. **(B)** Soluble monomeric Aβ38, Aβ40, and Aβ42 were quantified by ECLIA in the culture media of transfected cells. Values (means ± SEM) given in pg/ml are representative of three independent experiments (*n* = 3 in each experiment). ^∗∗∗^*p* < 0.001, ^∗^*p* < 0.05, as compared to control (mock-transfected cells).

As previously reported, βCTFs are barely detectable upon APP expression (**Figure 2**), the vast majority of APP being processed by the non-amyloidogenic pathway in cells (Haas et al., 1995). C99 expression (corresponding to the β-CTF) led to the detection of a monomeric band and the previously reported higher molecular weight band (**Figure 2A**). Strikingly, C42 expression produced a similar high molecular weight band, whereas no band corresponding to monomeric C42 was detected under these conditions. Of note, this higher molecular weight band was not detected in APP-expressing cell lysates. We previously suggested (Kienlen-Campard et al., 2008) that this band could correspond to C99 dimers, but the molecular weight (∼25–30 kDa) observed here is not consistent with this idea (**Figure 2A**). Importantly, the oligomeric band detected was not recognized by an antibody directed against the APP C-terminus that recognizes C99 and APP CTFs (**Figure 2A**). Sub-cellular fractionation experiments showed that the oligomeric band is enriched in vesicular compartments (MLP) but barely absent from the soluble fractions (**Figure 2B**), strongly suggesting that the oligomers -like C99- are either membrane associated, or present in a cell membrane-bounded compartment but not in the cytosol. The same oligomeric bands were revealed by Western blotting in the culture medium of cells expressing either C99 or C42 (**Figure 3A**), but they were not detectable in the medium of cells expressing full-length APP. We measured the production of soluble Aβ in the culture media by the highly sensitive ECLIA multiplex Aβ assay, which detects soluble Aβ38/Aβ40/Aβ42 monomeric isoforms in the same sample (Hage et al., 2013, 2015). As previously shown, soluble monomeric Aβ was readily detected in culture media of APP- and C99-expressing cells, with Aβ40 being the most abundant isoform. C99 constructs produced 3–5 times more Aβ than full length APP (**Figure 3B**). Strikingly, the C42 construct did not produce detectable Aβ, except very low amounts of Aβ38. Due to the specificity of the assay (soluble monomeric Aβ isoforms), this suggested that only oligomeric Aβ isoforms were generated by C42-expressing cells, in line with the higher molecular weight bands detected by an anti-human Aβ antibody in the same media (**Figure 3A**).

**FIGURE 4:**
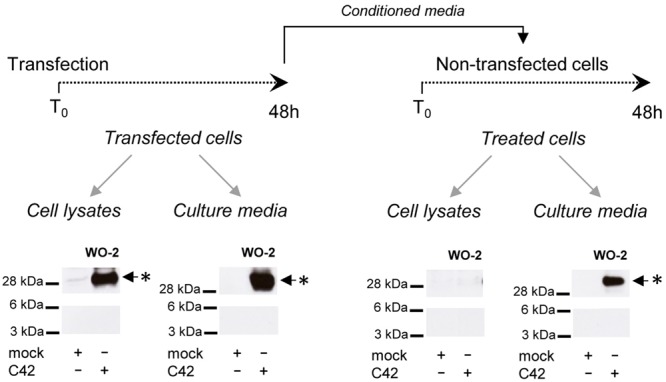
**Oligomers detected in cells and not re-captured from the culture medium.** CHO cells were either transfected by the C42 construct (transfected cells) or treated (treated cells) for 48 h with the conditioned culture medium of C42-transfected cells (mock, non-transfected), as depicted on the top of the figure. The cell lysates and culture media of transfected and treated cells were recovered and analyzed by Western blotting with the W0-2 antibody. The ∼30 kDa oligomer band (^∗^) detected in cell lysates and culture media is indicated by an arrow.

We next investigated whether the oligomeric bands detected in cells could correspond to oligomeric Aβ recaptured from the culture medium. We used conditioned media of C42-expressing cells to treat non-transfected cells for 48 h. Media and cells were collected and analyzed after treatment (**Figure 4**). The ∼30 kDa band was still present in the culture medium after treatment, indicating its stability. No similar signal was found in the lysates of treated cells, in contrast to those of C42-expressing cells. This indicated the oligomers detected were primarily produced in cells and not recaptured from the culture medium where they are also present.

Together, these results strongly suggested that the high MW band could correspond to oligomeric Aβ42 peptides, but did not formally exclude that they are formed by the assembly of other Aβ isoforms (e.g., the major Aβ40 isoform) or truncated APP C-terminal fragments. To test this hypothesis we expressed C40 constructs in cells, which correspond to the Aβ40 sequence. C40-expressing cells did not produce the oligomers found in C42-expressing cells (**Supplementary Figure S1**). This indicates that the oligomeric bands observed are produced by Aβ42- but not Aβ40-expressing constructs. To further confirm this hypothesis, bands around 30 kDa were excised from gels and subjected to nano liquid chromatography (nano-LC) coupled to tandem mass spectrometry (MS/MS). The peptides identified correspond to human Aβ sequences (**Supplementary Figure S2**). No other APP fragments (e.g., from the C-terminus) were identified in these gel samples, even in C99-expressing cells. Thus, we can conclude that the oligomers detected in transfected cells are indeed Aβ oligomers, and more precisely Aβ42 oligomers.

### Mutations of Glycine Residues Present in GXXXG on GXXXA Motifs Are Critical for the Aβ Oligomerization Process

**FIGURE 5:**
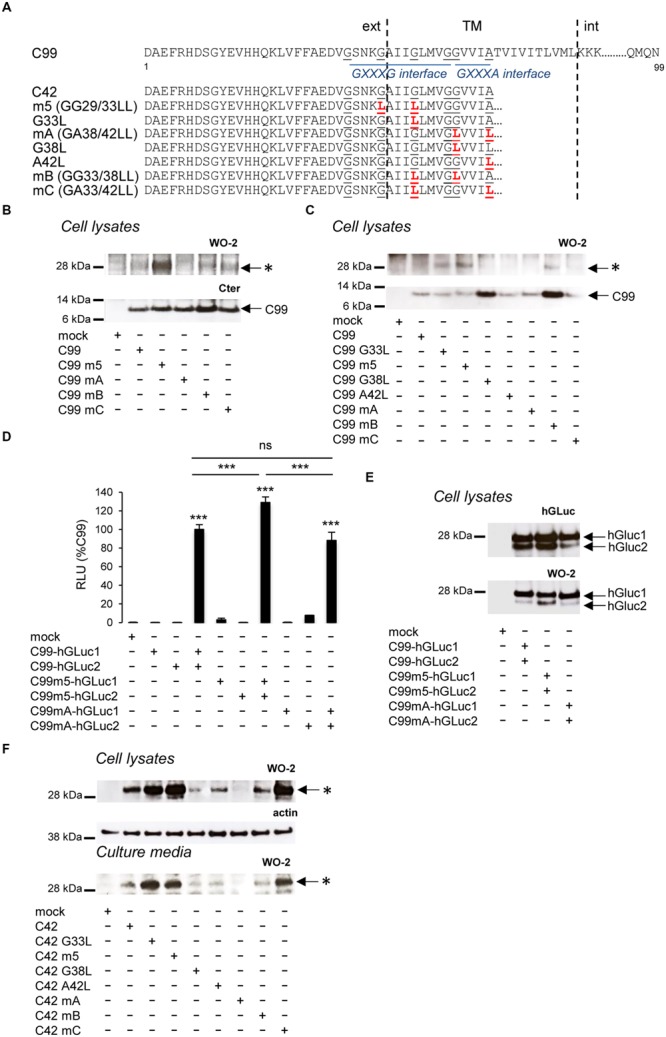
**Impact of GXXXG/GXXXA mutations on C99 dimerization and Aβ oligomerization. (A)** Schematic representation of human the C99 and C42 constructs used. Numbering corresponds to amino acid position in the C99 sequence. The amino acid substitutions generated by site-directed mutagenesis are in red. Glycine and alanine residues of GXXXG/GXXA sequences are underlined. TM, Transmembrane region; ext, extracellular; int, intracellular. **(B)** Expression of C99 and Aβ oligomers analyzed in cell lysates by Western blotting with the APP C-terminal antibody and the W0-2 antibody, respectively. Oligomers (^∗^) and C99 are indicated by arrows. **(C)** Expression of C99 and Aβ oligomers analyzed in lysates of cells expressing the different C99 mutants by Western blotting with the W0-2 antibody. Oligomers (^∗^) and C99 are indicated by an arrow. **(D,E)** Dimerization of C99 and C99 mutants (m5, mA) was measured in living cells by the split-luciferase complementation assay. Cells were transfected with C99-coding sequences fused to two hGLuc moieties (hGluc1 and 2, see **Figure 1**). Bioluminescence (luciferase activity) was measured as RLU and given as percentage of bioluminescence detected in cells co-expressing C99-hGLuc1 and C99-hGLuc2. Values (means ± SEM) are representative of three independent experiments (*n* = 4 in each experiment). ^∗∗∗^*p* < 0.001, n.s. (non-significant), as compared to control (mock-transfected cells). Expression of the fusion proteins was checked in cell lysates by Western blotting with the hGLuc antibody and the W0-2 antibody. **(F)** Analysis of Aβ oligomerization was monitored by Western blotting with the W0-2 antibody in cell lysates or culture media of cells expressing C42 or C42 mutants. Actin was used as loading probe (cell lysates). Oligomers (^∗^) are indicated by an arrow.

We previously reported that the oligomeric bands identified here as Aβ42 oligomers were more abundant using C99 constructs mutated in the GXXXG motifs (Kienlen-Campard et al., 2008). In our initial hypothesis, GXXXG mutations induced rotations in the TM helical regions that form stable associations through the GXXXA interface, strengthening dimerization of the APP transmembrane domain (TMD). We mutated the glycine and alanine residues within the G^29^XXXG^33^/G^38^XXXA^42^ motifs (**Figure 5A**) to study their contribution to C99 dimerization and Aβ oligomerization. The level of the Aβ oligomeric band was highly increased in cells expressing C99 mutated in the central GXXXG motif (GG29/33LL, referred to as m5) with respect to cells expressing non-mutated C99, but the size of the oligomers remained the same (**Figure 5B**). Strikingly, the GA38/42LL mutation (referred to as mA) abolished Aβ oligomer production, and mutations affecting G^29^XXXG^33^, G^33^XXXG^37^ and G^38^XXXA^42^ motifs (referred to as mB and mC) produced intermediate levels of oligomers. We analyzed the contribution to oligomer formation of key G/A residues in the G^29^XXXG^33^/G^38^XXXA^42^ motifs. The single G33L mutation was sufficient to induce the oligomerization profile observed with m5 (GG23/33LL). In that context (G33L), mutation of small residues from the G^38^XXXA^42^ interface (mB = GG33/38LL and mC = GA33/42LL) reduced the oligomerization promoted by the mutation of the critical G33 residue (**Figure 5C**). We found that these oligomeric bands did not result from changes in C99 dimerization (Munter et al., 2007; Kienlen-Campard et al., 2008; Ben et al., 2012a). Dimerization measured by a highly sensitive split protein assay (Decock et al., 2015) was found to be equivalent for C99, C99 m5, and C99 mA (**Figures 5D,E**), whereas oligomeric bands are abundant in C99 m5 but not in C99 mA cell lysates (**Figure 5B**). Altogether, this indicated that mutations of glycine residues could play a critical but dual role in Aβ production, without major changes in overall dimerization of C99. This idea was further addressed by expressing mutants of C42 in cells. Identical mutations to those studied in the C99 were introduced in C42 (**Figure 5A**). Mutation of glycine residues from the G^29^XXXG^33^ and G^33^XXXG^37^ motifs, especially the G33L mutation, strongly increased the production of Aβ oligomers, which migrated at the same size as C99-derived oligomers (**Figures 5B,C**). Mutation of small residues (G/A) of the G^38^XXXA^42^ motif abolished oligomerization (**Figure 5F**). Strikingly, the single G38L mutation decreased oligomerization of C42, and the G38L mutation in the context of G33L (mB) strongly impaired oligomerization of G33L mutants. On the other hand, the A42L mutation had moderate effects and was not able to counteract the increased oligomerization induced by G33L mutation (mC). The oligomers detected had the same electrophoretic profile in cell lysates and culture media, confirming the idea that oligomers are formed in cells and released into the culture medium. The critical residues for Aβ oligomerization are G33 and G38. G33L mutation strongly promotes oligomerization; G38L blocks it with a dominant effect on G33 residue modification.

### Oligomeric Aβ42 Peptides Affect Neuronal Differentiation

**FIGURE 6:**
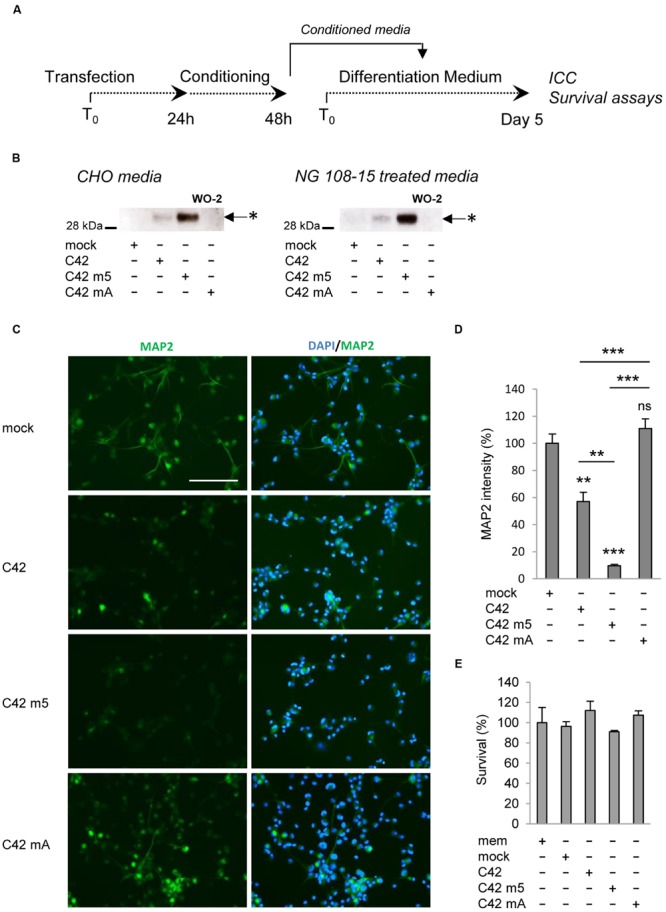
**Effects of Aβ oligomers on neuronal cell differentiation and survival. (A)** Neuronal NG108-15 cells were treated with media of control CHO cells (mock) or CHO cells transfected with C42, C42 mutant 5 (m5) or C42 mutant A (mA) during the differentiation process. **(B)** The presence of Aβ oligomers in the media of CHO producing cells and NG108-15 cells (at day 5) was assessed by Western blotting with the W0-2 antibody. Oligomers (^∗^) are indicated by arrows. **(C)** Neuronal differentiation was assessed by immunostaining of NG108-15 treated cells with the MAP2 antibody. Nuclei were stained with the DAPI. Scale: 200 μm. **(D)** Quantification of MAP2 immunostaining was performed and expressed as percentage of intensity measured in NG108-15 cells treated with the control medium (mock). Values (means ± SEM) are representative of three independent experiments (*n* = 2 in each experiment). ^∗∗∗^*p* < 0.0001, ^∗∗^*p* < 0.001, ns (non-significant) as compared to cells treated with the control medium. **(E)** Survival of the NG108-15 cells was assessed by MTS assay and given as percentage of survival measured in cells treated with the control medium. Values (means ± SEM) are representative of three independent experiments (*n* = 3 in each experiment). Statistical analysis showed non-significant differences between the conditions.

One important question was to understand whether the oligomers we found to be produced in living cells and released in the extracellular medium displayed pathological properties. A growing number of studies indicate that the pathological properties of Aβ are directly related to the formation of particular oligomeric assemblies (Benilova et al., 2012). We used the culture medium of CHO cells transfected with C42, C42m5, and C42mA to treat neuronal NG108-15 cells when differentiation is induced (**Figure 6A**). Culture media recovered from transfected CHO cells (after 24 h of conditioning) showed the Aβ oligomeric profile detailed above: oligomeric Aβ was found in C42-expressing cells, at higher levels in cells expressing C42m5, but not in those expressing C42mA. All had the same electrophoretic profile. NG108-15 cells were treated for 5 days with CHO cell medium, renewed every 48 h. At the end of the treatment, oligomeric bands of Aβ were readily detectable in medium of treated NG108-15 cells (**Figure 6B**). Five days after treatment, control cells showed the typical neurite outgrowth of differentiated NG108-15 cells (Stanga et al., 2015) measured by MAP2 staining (**Figures 6C,D**). NG108-15 cells treated with medium from C42- and C42m5-expressing CHO cells showed altered differentiation and reduced neurite outgrowth. The intensity of MAP2 staining was particularly reduced in cells treated with media from C42m5-expressing cells, in which the highest concentration of Aβ oligomers was detected. In contrast, the differentiation pattern of cells treated with media of C42mA-expressing cells, in which no oligomeric Aβ is detected, was comparable to that of non-treated cells. Thus, differentiation and neurite outgrowth of NG108-15 cells is impaired by the presence of Aβ oligomers. Importantly, this is not simply linked to cytotoxicity of these oligomers since the treatments did not affect NG108-15 cell survival in all the conditions tested (**Figure 6E**). Together, these data highlight an intrinsic property of specific Aβ oligomers to disrupt the formation of a neuronal network, which might be a very early event in β-amyloid pathologies.

## Discussion

Our major findings are as follows: (i) expression of a construct coding for a peptide corresponding to Aβ42 leads to the formation of oligomers of 28–30 kDa (with no detection of monomeric Aβ42) that are resistant to denaturation and display neuropathological properties; (ii) the same size oligomer can be detected when C99 (β-CTF) is expressed, but in this case a monomeric form can also be detected, suggesting that the location of processing in the cell can influence the extent of oligomerization; (iii) Aβ40 does not induce the formation of such oligomeric species; (iv) the oligomers induced by Aβ42 and C99 are similar, and their size remains identical upon mutation; (v) the formation of oligomers is regulated by the G^29^XXXG^33^ and G^38^XXXA^42^ transmembrane motifs – G33 of the G^29^XXXG^33^ motifs exerts a negative effect and G38 of the GXXXA motif exerts a stimulatory effect on oligomer formation; and (vi) the same motifs also regulate processing of C99 (Kienlen-Campard et al., 2008) without affecting the extent of C99 dimerization in cells.

### Aβ42 Oligomers Are Formed in Cells

The oligomeric peptides revealed by Western blotting that were initially thought to be C99 dimers (Kienlen-Campard et al., 2008) correspond indeed to Aβ42 oligomers. These Aβ oligomers are produced in cells either by the processing of the APP amyloidogenic fragment (C99) to Aβ, or when a peptide corresponding to Aβ42 is expressed in cells. Strikingly, soluble monomeric Aβ forms are only detected in C99-expressing cells and not when Aβ42 is expressed. Thus, the maintenance of soluble Aβ42 might depend on intracellular compartments where C99 fragments are processed. C42 fragments generate Aβ42 without being processed by the γ-secretase. We found that C55 fragments containing the KKK capping motifs at the TM/intracellular junction become substrates for γ-secretase (**Supplementary Figure S4**). This observation is quite intriguing and needs to be further investigated, but it suggests that monomeric Aβ can only be produced by specific substrates and in specific compartments, with the short sequence spanning residues 42–55 being sufficient to localize it to a compartment productive for γ-secretase cleavage. As soon as these requirements are met, Aβ shows a high propensity to aggregate, both in cells and in the supernatant. When Aβ42 is directly produced in cellular compartments (C42 constructs), it spontaneously aggregates to form oligomers; soluble monomeric Aβ is no longer detectable. In this regard, it remains to be determined whether co-expression of Aβ42 and C99 would allow the detection of soluble Aβ42, would increase the levels of Aβ42 oligomers or promote formation of a higher molecular weight oligomer. The Aβ oligomers we detected are enriched in membrane-bound compartments and found in cell culture medium in conditions where no cytotoxicity is readily measured in CHO cells. They are thus secreted rather than simply released in the extracellular space upon cell death and subsequent leaking of intracellular components. In addition, we found that intracellular oligomers are not recaptured from the culture medium, or at least at very low levels that cannot be detected in our experimental conditions. This observation strongly supports the conclusion that Aβ oligomers are readily formed in cells and further secreted in the extracellular medium. Intriguingly, only Aβ assemblies of ∼25/30 kDa, corresponding to the expected molecular weight of Aβ hexamers, can be detected under our experimental conditions. It does not exclude that other Aβ oligomers are produced by the cells, but their conformation should in this case not be stable enough to withstand with the high temperature denaturing conditions (SDS) we used to run Western blots. The Aβ oligomers we identified are very stable, resistant to SDS, temperature (**Supplemental Figure S3**) and formic acid denaturation (not shown).

Oligomers were detected only with constructs expressing Aβ42, and not Aβ40. Aβ42, unlike Aβ40, can form stable trimeric and tetrameric complexes (Chen and Glabe, 2006) as well as globulomer structures (dodecamers) in the presence of SDS (Barghorn et al., 2005), and can form pentamers and hexamers in solution (Bitan et al., 2003; Bernstein et al., 2009). SDS was suggested to artificially produce stable Aβ assemblies. We found Aβ oligomers in the culture media that were not isolated by SDS-based extraction, suggesting that they are not generated by a spurious effect of the detergent. One obvious question is to understand how such Aβ42 oligomers can assemble in cells. Previous studies showed that low-temperature and low-salt conditions can stabilize disk-shaped oligomers (Ahmed et al., 2010). They do not have the β-sheet structure characteristic of fibrils, but are rather composed of loosely aggregated strands. Importantly, it has recently been shown that at physiological temperature (37°C) and at high concentration, Aβ42 rapidly aggregates into unstructured specific oligomers (Fu et al., 2015). The high concentrations achieved here by direct expression of Aβ42 could indeed mimic pathological situation in which Aβ accumulates in specific cellular compartments.

### G33 and G38 Residues from G^29^XXXG^33^ and G^38^XXXA^42^ Motifs Dramatically Affect the Aβ Oligomerization Process

Small amino acid residues (G/A) present in GXXXG or GXXXA motifs are known to promote TM helix association. Their mutation was shown to strongly affect the processing of the β-CTF (Munter et al., 2007; Kienlen-Campard et al., 2008). Using a protein fragment complementation assay based on *Gaussia princeps* luciferase, we show that the effects on C99 dimerization of mutating the G^29^XXXG^33^ and G^38^XXXA^42^ motifs are not major. While these motifs might change the precise interface of dimers, they do not control the overall extent of dimerization, at least when measured by the proximity of the C-terminal ends. These results are in line with our recently published work (Decock et al., 2015) showing that the intracellular domain of C99 has a dominant influence on C99 dimerization. GXXXG motifs present in APP TM region might regulate processing of amyloidogenic CTFs either independently of dimerization or by controlling precise dimeric conformations.

We showed that mutation of these glycine residues indeed dramatically affected the formation of Aβ oligomers. G33 and G38 play a predominant role in this process by promoting or blocking oligomerization, respectively. They belong to two different motifs composed of small residues G/A separated by three aminoacids, with G38 being like a pivot. Importantly, identical Aβ oligomers were detected in cells expressing mutated or non-mutated C99 or C42. For the GXXXG mutants, high amounts of oligomers were detected, but no soluble monomeric Aβ is measured by ECLIA. Soluble monomeric Aβ was not detected for the different GXXXA mutants, but the mutations in these constructs are at positions preventing detection by the C-terminal ECLIA capture antibody. Our results would suggest that soluble monomeric Aβ is produced by the different mutants, but that the mutations shift the monomeric-oligomeric Aβ equilibrium toward the formation of oligomeric Aβ forms (G33 mutants), or block the conversion from monomers to oligomers (G38 mutants). The turn in the Aβ structure at G38 is a characteristic of Aβ42 oligomers and molecular contacts have been reported in the monomeric unit of Aβ42 fibrils between G38 and F19 (Luhrs et al., 2005) and between M35 and A42 (Masuda et al., 2008). The particular roles of G38 and A42 could first explain the low oligomerization profile of Aβ produced from C99mA and C42mA in which both G38 and A42 are mutated, and the absence of oligomer formation with Aβ40 lacking the A42 position. G38 is important to form a solvent accessible turn in the β-hairpin structure of the Aβ42 building blocks assembled in oligomers. G38 to L mutation could thus break the conformation of Aβ monomers which promotes the formation of oligomers as proposed by Ahmed et al. (2010). G33 residue is part of the G^33^LMVG^37^ hydrophobic sequence forming a β-strand which is critical for the Aβ monomers to adopt an antiparallel β-hairpin structure present in Aβ oligomers prior to conversion to β-sheet structure. Mutation of G33 is thus very likely to modify Aβ conformation in a way that favors oligomerization. Our results are in line with previous studies highlighting G33 as critical for the generation of Aβ42 assemblies (Harmeier et al., 2009). Mutation of G33 was suggested to promote rapid Aβ oligomerization by conformational changes favoring the formation of high molecular weight oligomers. However, these observations await further structural studies in order to clearly understand how G33 and G38 can contribute to Aβ oligomerization in opposite ways as we observed in cells.

### Aβ42 Oligomers Produced by Cells Display Neuropathological Properties

Emerging evidence indicates that Aβ oligomers, and not Aβ fibrils, have neuropathological properties. Aβ oligomers can rapidly interact with cell membranes and display neurotoxic effects, before a further possible conversion to protofibrils or fibrils (Ahmed et al., 2010). Apart from cytotoxic effects, Aβ oligomers have been shown to cause various neuronal dysfunctions. Accumulation of soluble (non-fibrillar) Aβ forms is correlated with the progression of AD (McLean et al., 1999) and is a predictor of synaptic changes and disruption of neuronal circuits occurring in the pathology (Hsia et al., 1999; Lue et al., 1999). We showed here that Aβ oligomers released in the cell medium strongly impair neuronal differentiation and neurite outgrowth, but did not display significant neurotoxicity. The neurotoxic effect of Aβ42 was reported to depend on its intraneuronal accumulation (Kienlen-Campard and Octave, 2002; Bayer and Wirths, 2010), but this is restricted to neurons or primary neuron cultures and does not occur in cell lines (Kienlen-Campard et al., 2002). Interestingly, we observed intracellular accumulation of Aβ oligomers in cell lines (CHO) without observing cytotoxic effects. It is very likely that Aβ-related cytotoxicity takes place in differentiated post-mitotic neurons, by specific yet poorly understood mechanisms that could involve autophagy. It would be therefore of particular interest to further address Aβ42 oligomer formation, distribution and cytotoxicity in neurons or neuronal cultures. More specifically, it would be useful to decipher in neuronal model if the toxicity related to intracellular Aβ accumulation involves oligomers, and the precise mechanisms underlying the pathological properties of extracellular Aβ assemblies. Our results do not exclude that long-time exposure to Aβ oligomers we identified could trigger toxic effects *per se*. One could also imagine that pathological effects of Aβ oligomers involve specific receptors, among which APP itself (Lorenzo et al., 2000). This could be evaluated in APP deficient (KO) mice models (Heber et al., 2000). All these processes, which need to be further elaborated, could be highly relevant for sporadic Alzheimer’s disease where with age the capacity to maintain soluble Aβ decreases and Aβ42 accumulates in neurons as an early event. Previous studies have shown that Aβ concentrated by more than two orders of magnitude can reach micromolar concentrations in acidic vesicular compartments (Hu et al., 2009). This local high increase in Aβ concentration could initiate oligomerization in these compartments. Together these observations provide a strong impetus to address the formation and precise subcellular localization of Aβ oligomers in neurons, and to analyze whether and how they are related to neuronal dysfunction.

However, the fact that Aβ oligomers did not induce cytotoxic effects, but strongly impaired neurite outgrowth and maturation of the neuronal network is in line with previous observations. The extracellular accumulation of a 56-kDa soluble Aβ oligomer, isolated from brain of AD transgenic mice, impaired memory independently of any neuronal loss (Lesne et al., 2006). It would be of particular interest to investigate if oligomers identical to the ones we identified here can be isolated from transgenic AD mice or brains of AD patients. The role of these oligomers on the formation of neurofibrillary tangles (NFTs), another key AD lesion, should also be addressed. Indeed, amyloid deposition has been shown to dramatically aggravate tauopathy in AD mice models (Stancu et al., 2014). It is also important to note that neuronal-specific mechanisms, like post-translational modifications, can participate in Aβ oligomerization. For instance, phosphorylation at different positions, including Ser8 and Ser26, have been shown to enhance or block the formation of Aβ oligomers in brains (Kumar et al., 2011, 2013). Whether these neuronal mechanisms regulate the Aβ assemblies we identified here should be further explored. Up until now, a puzzling list of Aβ oligomer species supposed to be multimers of a fundamental Aβ trimer assembly (hexamers, nonamers, and dodecamers) have been identified from various experimental systems (including human AD brains and brains of AD transgenic mice) and isolated under different experimental conditions (Benilova et al., 2012). Still, they provide no clear evidence as to which are pathologically relevant, and the molecular mechanisms underlying their precise assembly are poorly understood.

## Conclusion

We reported here that specific Aβ oligomeric forms can be produced in living cells, and secreted into the extracellular medium. Such Aβ assemblies are stable, resistant to temperature and SDS denaturation, and impair neuronal maturation and differentiation. They are detected when Aβ is generated upon C99 processing, and are identified at high levels when Aβ42, but not Aβ40, is directly expressed in cells. We found two glycine residues, G33 and G38, to be critical in Aβ assembly into this oligomer form. Mutation of G33 dramatically increases Aβ oligomerization, whereas mutation of G38 impairs it. These observations suggest a protective role and a triggering role for G33 and G38 in the Aβ oligomerization process, respectively, and are in line with previous studies highlighting the important role of the G38 in the Aβ strand-turn-strand conformation specifically observed in oligomers. In conclusion, our data provide experimental evidence that Aβ oligomers observed *in vitro* are readily produced in membranes of living cells, and prompt further investigation into their precise structure, how they assemble, and their pathological relevance to human disease.

## Author Contributions

MD and PK-C designed research. MD and SS conducted experiments. MD, SS, SC, and PK-C analyzed data and wrote the paper with fundamental input of SOS, ID, and J-NO.

## Conflict of Interest Statement

The authors declare that the research was conducted in the absence of any commercial or financial relationships that could be construed as a potential conflict of interest.

## Supplementary Material

FIGURE S1 **Expression of C40 and C42 mA do not lead to Aβ oligomers formation.** Cells were transfected with the control empty vector (mock), or constructs expressing C42 or C40. **(A)** Analysis of C42/C40 expression and Aβ oligomerization was monitored in cell lysates by Western blotting with the W0-2 antibody. Oligomers (^∗^) are indicated by arrows. **(B)** Expression of C42 and C40 mRNA levels were measured by RTqPCR with the same primers and given as percentage of mRNA levels measured in C42-expressing cells.

FIGURE S2 **Mass spectrometry analysis of the oligomeric bands produced in C42-expressing cells.** Cell lysates from non-transfected (mock) and C42 transfected cell were separated on NuPage gradient gels in denaturating conditions. Gel bands around 30 kDa were excised and proteins were extracted and digested with trypsin. Following digestion, purified peptide samples were analyzed by nanoLC coupled to tandem mass spectrometry (MS/MS). The raw MS file were analyzed and searched against the APP protein sequence database. The results summarized showed the high confidence identification of two peptide sequences contained in the C42/Aβ42 peptides. C42 and Aβ42 sequences are given on the top of the tables. Peptide sequences identified are in bold. Signal peptide sequence is in blue.

FIGURE S3 **Resistance of Aβ oligomers to temperature.** Media of cells expressing C42 or C42m5 were collected and heated at 95°C for 0, 10 to 30 min prior to Western blotting revealed with the W0-2 antibody. Oligomers (^∗^) are indicated by an arrow.

FIGURE S4 **Aβ oligomers formation in cells expressing different C-terminal truncations of C99. (A)** Schematic representation of the different constructs. C99 corresponds to the APP β C-terminal fragment. Numbering corresponds to aminoacid position in the C99 sequence. C55, C49, and C42 have been generated by entering a stop codon at positions 55, 49, and 42 of C99, respectively. TM, Transmembrane region; ext, extracellular; int, intracellular. The aminoacid substitution (referred to as m5) generated for each construct appears in bold and red. **(B)** Expression of C99, C99 m5, C45 m5, C49 m5, and C55 m5 in CHO cells analyzed by Western blotting with the W0-2 antibody. Oligomers (^∗^) and monomers are indicated by arrows. **(C)** Aβ 38, 40, and 42 were quantified by ECLIA in the culture media of transfected cells. Values (means ± SEM) given in pg/ml are representative of three independent experiments (*n* = 3 in each experiment). ^∗^*p* < 0.05, ^∗∗∗^*p* < 0.001, as compared to control cells (mock-transfected cells).
