# Supplementary figures and images for: Glycines from the APP GXXXG/GXXXA Transmembrane Motifs Promote Formation of Pathogenic Aβ Oligomers in Cells
Source: Front Aging Neurosci. 2016 May 10;8:107. doi: 10.3389/fnagi.2016.00107 (PMC4861705; doi:10.3389/fnagi.2016.00107)

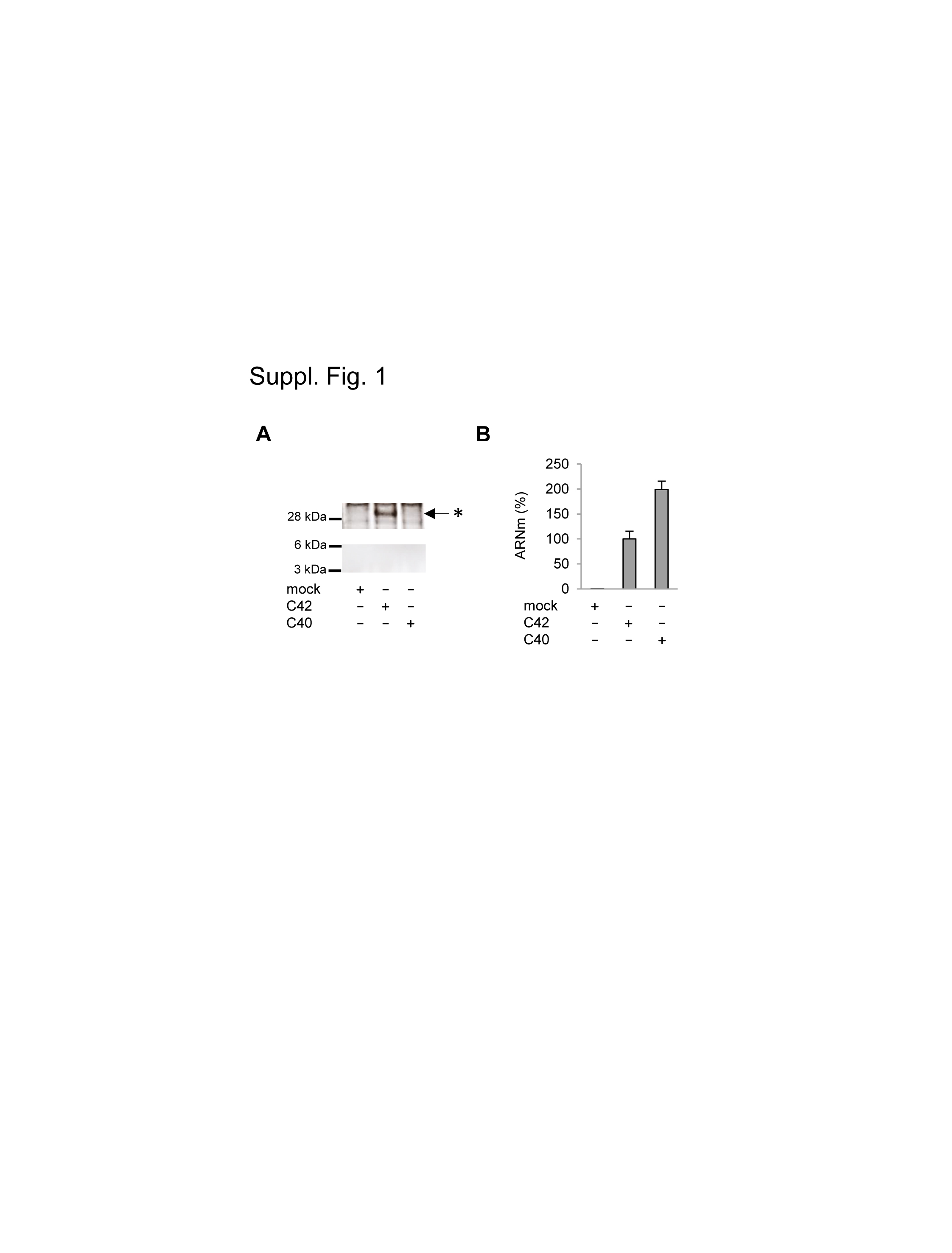

Supplement: FIGURE S1 — Expression of C40 and C42 mA do not lead to Aβ oligomers formation. Cells were transfected with the control empty vector (mock), or constructs expressing C42 or C40. (A) Analysis of C42/C40 expression and Aβ oligomerization was monitored in cell lysates by Western blotting with the W0-2 antibody. Oligomers (∗) are indicated by arrows. (B) Expression of C42 and C40 mRNA levels were measured by RTqPCR with the same primers and given as percentage of mRNA levels measured in C42-expressing cells. [file Image_1.JPEG]

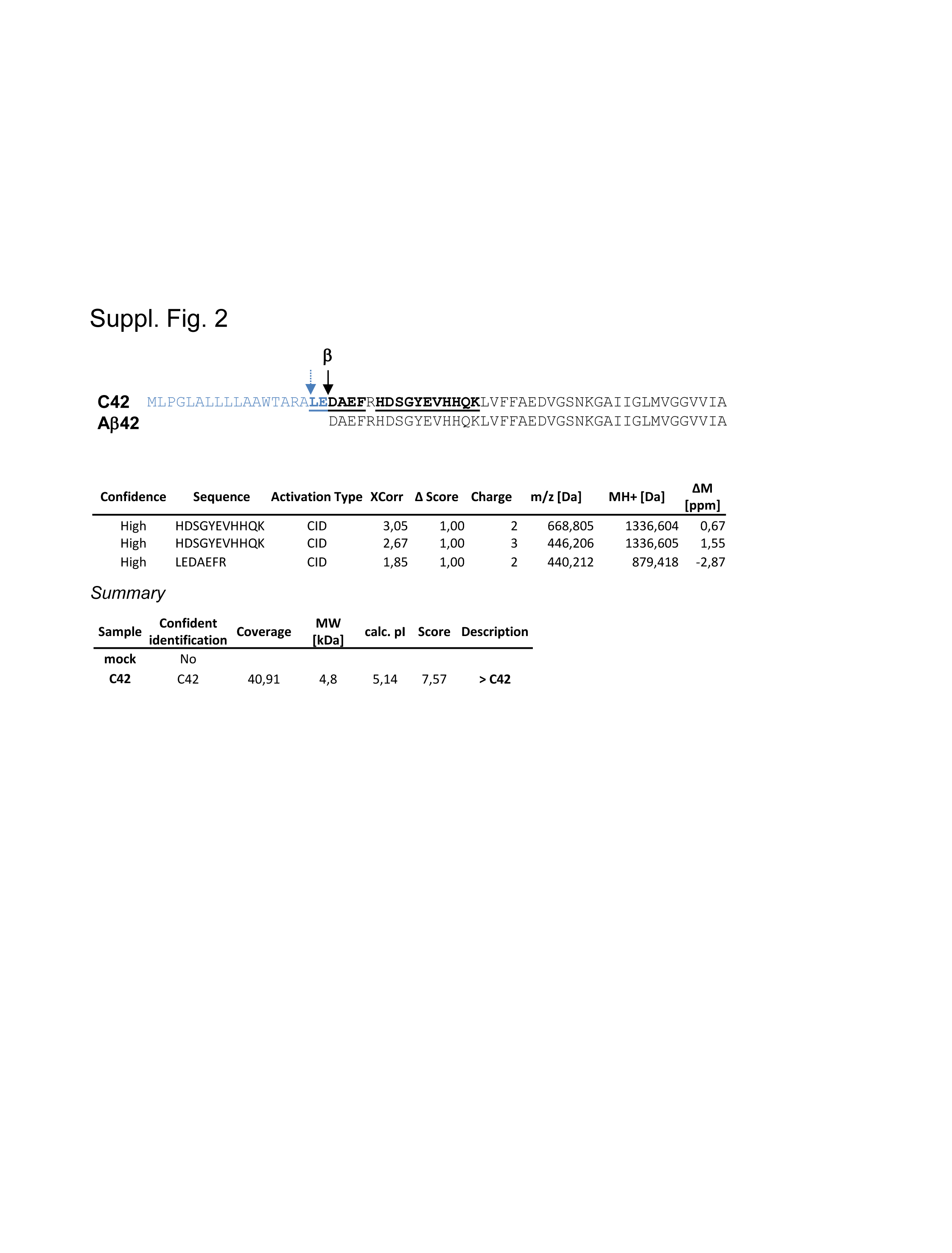

Supplement: FIGURE S2 — Mass spectrometry analysis of the oligomeric bands produced in C42-expressing cells. Cell lysates from non-transfected (mock) and C42 transfected cell were separated on NuPage gradient gels in denaturating conditions. Gel bands around 30 kDa were excised and proteins were extracted and digested with trypsin. Following digestion, purified peptide samples were analyzed by nanoLC coupled to tandem mass spectrometry (MS/MS). The raw MS file were analyzed and searched against the APP protein sequence database. The results summarized showed the high confidence identification of two peptide sequences contained in the C42/Aβ42 peptides. C42 and Aβ42 sequences are given on the top of the tables. Peptide sequences identified are in bold. Signal peptide sequence is in blue. [file Image_2.JPEG]

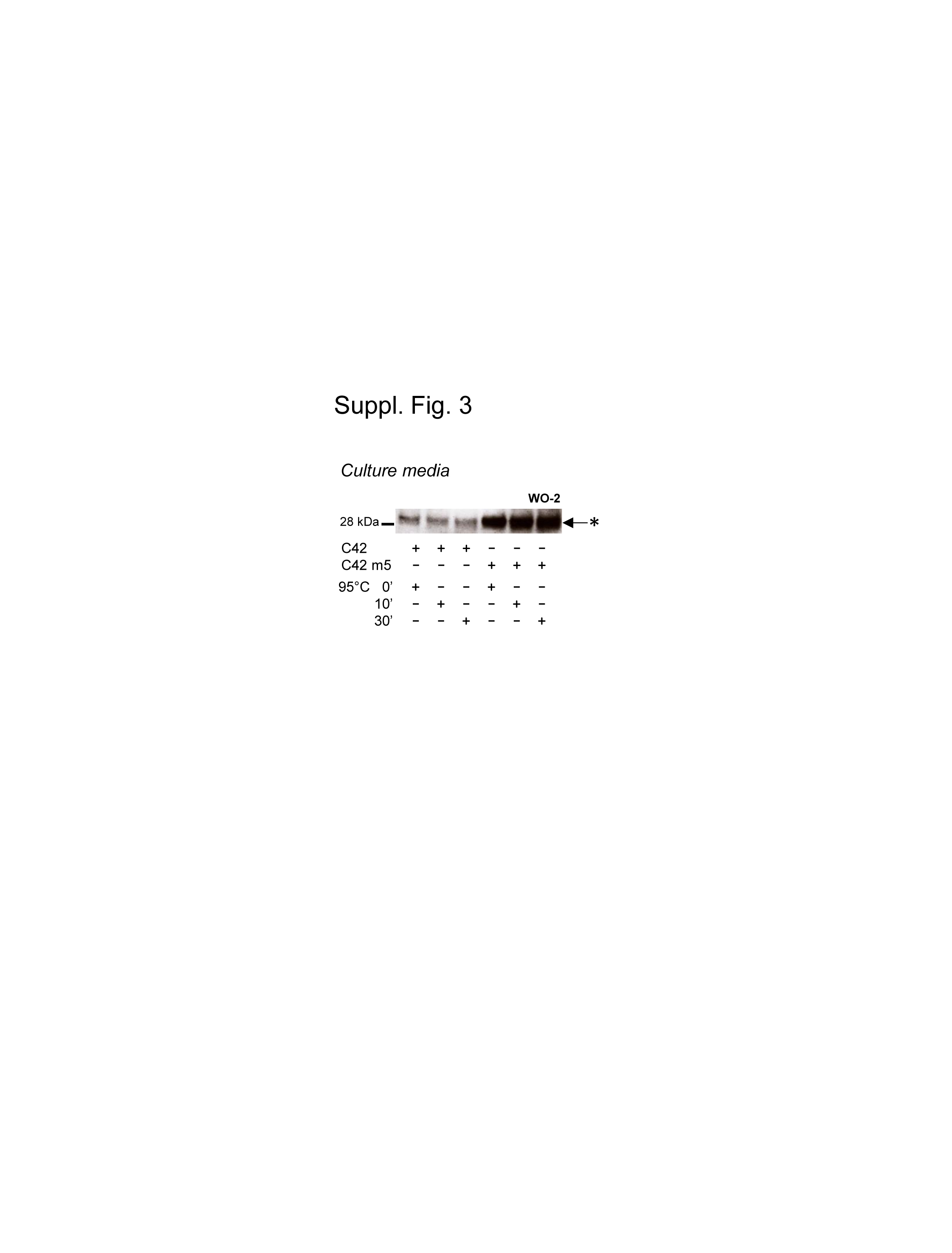

Supplement: FIGURE S3 — Resistance of Aβ oligomers to temperature. Media of cells expressing C42 or C42m5 were collected and heated at 95°C for 0, 10 to 30 min prior to Western blotting revealed with the W0-2 antibody. Oligomers (∗) are indicated by an arrow. [file Image_3.JPEG]

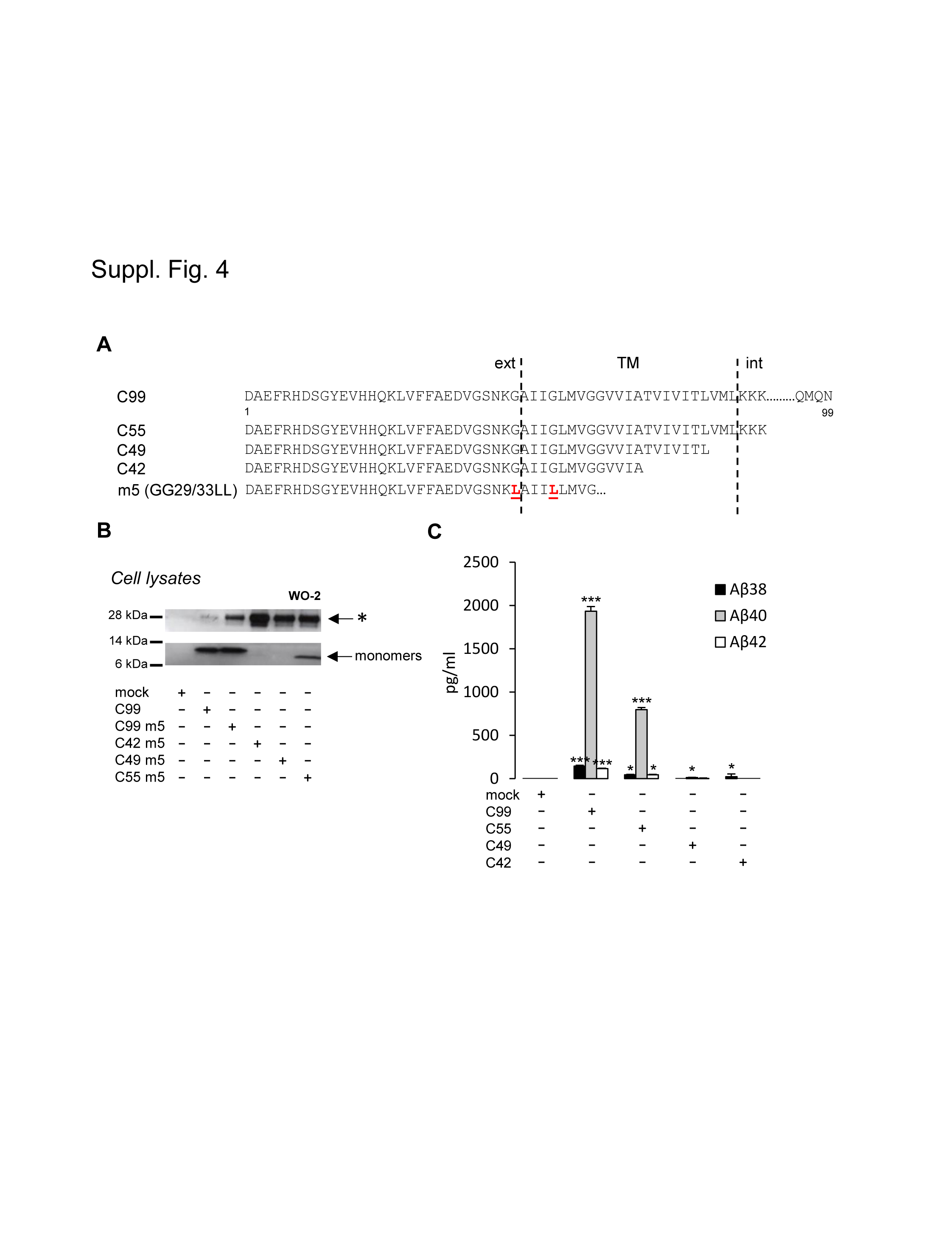

Supplement: FIGURE S4 — Aβ oligomers formation in cells expressing different C-terminal truncations of C99. (A) Schematic representation of the different constructs. C99 corresponds to the APP β C-terminal fragment. Numbering corresponds to aminoacid position in the C99 sequence. C55, C49, and C42 have been generated by entering a stop codon at positions 55, 49, and 42 of C99, respectively. TM, Transmembrane region; ext, extracellular; int, intracellular. The aminoacid substitution (referred to as m5) generated for each construct appears in bold and red. (B) Expression of C99, C99 m5, C45 m5, C49 m5, and C55 m5 in CHO cells analyzed by Western blotting with the W0-2 antibody. Oligomers (∗) and monomers are indicated by arrows. (C) Aβ 38, 40, and 42 were quantified by ECLIA in the culture media of transfected cells. Values (means ± SEM) given in pg/ml are representative of three independent experiments (n = 3 in each experiment). ∗p < 0.05, ∗∗∗p < 0.001, as compared to control cells (mock-transfected cells). [file Image_4.JPEG]
